# Serum and Fecal Amino Acid Profiles in Cats with Chronic Kidney Disease

**DOI:** 10.3390/vetsci9020084

**Published:** 2022-02-17

**Authors:** Stacie C. Summers, Jessica Quimby, Amanda Blake, Deborah Keys, Joerg M. Steiner, Jan Suchodolski

**Affiliations:** 1 Carlson College of Veterinary Medicine, Oregon State University, Corvallis, OR 97331, USA; 2 Department of Veterinary Clinical Sciences, The Ohio State University, Columbus, OH 43210, USA; quimby.19@osu.edu; 3 Texas A&M Gastrointestinal Laboratory, Department of Small Animal Clinical Sciences, College Station, TX 77843, USA; ablake@cvm.tamu.edu (A.B.); jsteiner@cvm.tamu.edu (J.M.S.); jsuchodolski@cvm.tamu.edu (J.S.); 4 Kaleidoscope Statistics Veterinary Medical Research Consulting, Athens, GA 30606, USA; deborah.a.keys@kaleidoscopestatistics.com

**Keywords:** cats, chronic kidney disease, amino acid, tryptophan

## Abstract

The purpose of the study was to quantify serum and fecal amino acids (AA) in cats with chronic kidney disease (CKD) and compare to healthy cats. Thirty-five cats with International Renal Interest Society Stage 1–4 CKD and 16 healthy mature adult and senior client-owned cats were included in this prospective cross-sectional study. Sera were analyzed for 25 AA concentrations using an ion exchange chromatography AA analyzer with post column ninhydrin derivatization. Voided fecal samples were analyzed for 22 AA concentrations using liquid chromatography with tandem mass spectrometry. CKD cats had lower serum concentrations of phenylalanine (mean difference ± standard error of the mean: 12.7 ± 4.3 µM; *p* = 0.03), threonine (29.6 ± 9.2 µM; *p* = 0.03), tryptophan (18.4 ± 5.4 µM; *p* = 0.005), serine (29.8 ± 12.6 µM; *p* = 0.03), and tyrosine (11.6 ± 3.8 µM; *p* = 0.01) and higher serum concentrations of aspartic acid (4.7 ± 2.0 µM; *p* = 0.01), β-alanine (3.4 ± 1.2 µM; *p* = 0.01), citrulline (5.7 ± 1.6 µM; *p* = 0.01), and taurine (109.9 ± 29.6 µM; *p* = 0.01) when compared to healthy cats. Fecal AA concentrations did not differ between healthy cats and CKD cats. 3-Methylhistidine-to-creatinine did not differ between healthy cats with and without muscle loss. Cats with CKD IRIS Stages 1–4 have a deranged serum amino acid profile compared to healthy cats.

## 1. Introduction

Muscle loss, with or without concurrent loss of fat stores, occurs in cats with chronic kidney disease (CKD) and can contribute to weight loss [1,2]. Cachexia is the loss of lean body mass in acute and chronic disease and is caused by the utilization of amino acids (AA) from muscle as a primary energy source [3]. Cachexia secondary to CKD has been best defined in people, with extrapolation to veterinary species. According to studies in people and rat models, cachexia is the result of negative energy intake, and increased protein catabolism secondary to metabolic acidosis, systemic inflammation, and insulin resistance [4]. In addition, CKD may cause protein malassimilation as a result of a decrease in the efficiency of protein digestion and decreased AA absorption in the small intestine [5,6]. Impaired protein digestion increases the abundance of proteolytic bacteria and fermentation of AAs to precursors of uremic toxins [4,7,8].

Amino acids are needed to synthesize proteins. There are 20 proteinogenic AAs, 11 of which are essential in cats. Compared to nonessential AAs, essential AAs cannot be synthesized by the organism and require dietary intake. Humans, dogs, and cats with kidney disease, even those with early disease, were previously reported to have a deranged profile of circulating AAs [9,10,11,12,13,14]. In addition, fecal AA profiles were shown to be altered in people receiving hemodialysis and in a rodent CKD model when compared to healthy controls [6,15]. To date, fecal AA concentrations and the correlation between serum and fecal AA concentrations in cats with CKD has not been documented.

An amino acid can undergo post-translational modifications, such as phosphorylation or methylation, to form closely related bioactive molecules. One such amino acid is 3-methylhistidine (3-MH), which is synthesized by skeletal muscle, released into circulation during muscle degradation, and excreted unchanged in the urine [16]. A previous study reported plasma 3-MH concentrations to be higher in cats with CKD compared to those in healthy control cats, and also in inappetent CKD cats compared to those with a normal appetite [9]. Age is associated with loss of lean body mass in cats in the absence of disease (i.e., sarcopenia), and senior cats more commonly have sarcopenia than young cats [17,18]. Because serum creatinine is a surrogate marker of skeletal muscle mass, normalizing 3-MH to creatinine concentrations (3-MH/Crea) has been used as a biomarker of muscle protein turnover in the elderly [19,20]. In veterinary medicine, the potential relationship between circulating 3-MH concentrations and muscle loss in cats has not previously been evaluated.

We hypothesized that cats with CKD would have a deranged serum and fecal AA profile when compared to healthy cats. In particular, we hypothesized that CKD cats would have reduced serum concentrations of essential AAs with concurrent increased fecal concentrations of those AAs, supporting protein malassimilation in the small intestine. In addition, we hypothesized that the 3-MH/Crea ratio would aide in the assessment of muscle loss in healthy mature adult and senior cats. To test these hypotheses, the primary objective of the study was to quantify serum and fecal AA concentrations in cats with CKD and compare them to those in a group of healthy cats. The secondary objective was to investigate the cross-sectional relationship of 3-MH/Crea with muscle condition score (MCS) in healthy mature adult and senior cats.

## 2. Materials and Methods

### 2.1. Study Design and Selection of Cats

In this prospective cross-sectional study, healthy mature adult and senior cats (≥8 years) and cats diagnosed with CKD were recruited from clients of the Colorado State University Veterinary Teaching Hospital between 2018 and 2020. To be eligible for inclusion, cats underwent a thorough evaluation that included a client history and review of the past medical record, complete physical examination performed by a single board-certified internal medicine specialist, CBC, serum biochemistry profile, urinalysis, serum total thyroxine concentration, blood pressure by Doppler, and urine protein-to-creatinine ratio (if the urine protein dipstick ≥1+). Physical examination included a 9-point body condition score (BCS; Nestle Purina, St. Louis, MO, USA) and muscle condition score (MCS) [21,22]. Cats with CKD were staged based on International Renal Interest Society (IRIS) guidelines [23]. Cats were staged as IRIS CKD Stage 1 based on serum creatinine <1.6 mg/dL with an inadequate urinary concentrating ability (urine specific gravity (USG) ≤1.035) and either abnormal renal palpation or renal imaging findings consistent with chronic renal degenerative disease. Cats were diagnosed as IRIS CKD Stage 2–4 based on a serum creatinine >1.6 mg/dL with an inadequate urinary concentrating ability. Cats were considered healthy based on an unremarkable client history, physical examination, and normal laboratory testing including a serum creatinine ≤1.8 mg/dL and urine specific gravity (USG) >1.035. At enrollment, owners were asked to subjectively assess the appetite of their cat using a 5-point scale (0%, 25%, 50%, 75%, or 100% of the ration consumed). 

Exclusion criteria included complications of CKD, such as acute obstructive urinary disease, urinary tract infection, or recent hospitalization, and a diagnosis of systemic disease including diabetes mellitus, hyperthyroidism, or known or suspected gastrointestinal disease (including food-responsive chronic enteropathy). 

### 2.2. Serum Amino Acid Analysis

Owners were instructed to withhold food from their cat for at least 12 h prior to blood collection. Blood was collected in sterile non-heparinized tubes and after formation of a clot centrifuged (within 30 min of sample collection) at 5000 rpm for 5 min. Serum was immediately harvested and stored at −80 °C for 0–2 weeks before being shipped on dry ice to Texas A&M Gastrointestinal Laboratory for analysis. Upon arrival, samples were thawed at room temperature and deproteinized with an equal volume of 5% (*w*/*v*) sulfosalicylic acid with 500 µM L-norleucine as an internal standard. Samples were then stored at 4 °C for 10 min prior to centrifugation at 10,000× *g* for 5 min at 4 °C. The supernatant was transferred to a PVDF 0.2 µm pore size centrifugal filter and centrifuged at 10,000× *g* for 5 min at 4 °C. Samples were then analyzed for amino acid content using an ion exchange chromatography amino acid analyzer with post column ninhydrin derivatization (Biochrom 30+, Biochrom Ltd., Holliston, MA, USA). One hundred microliters of the filtered supernatant was transferred to autosampler vials and stored at 4 °C for a maximum of 48 h prior to automated injection of 30 µL onto the analytical column.

Serum concentrations of the following 25 amino acids and related compounds were analyzed: β-alanine, L-alanine, L-arginine, L-asparagine, L-aspartic acid, L-citrulline, L-glutamic acid, L-glutamine, glycine, taurine, L-histidine, hydroxy-L-proline, L-isoleucine, L-leucine, L-lysine, L-methionine, 3-MH, L-ornithine, L-phenylalanine, L-proline, L-serine, L-threonine, L-tryptophan, L-tyrosine, and L-valine. The R2 of standard curves ranging from 2.5 µM to 750 µM were greater than 0.998 for all compounds. Peak area of the internal standard L-norleucine at 250 µM was within 2 standard deviations of the mean for all samples and standards. Working standard calibrator was prepared at 250 µM and was run every 10 injections with variation between resultant concentrations of less than 5%. Lower limits of quantification were calculated using the lowest concentration on the standard curve for which the observed to expected ratio remained between 80–120%. 

Amino acid concentrations were calculated using Biochrom BioSys V.3.0 software integrated with the EZChrom Elite™ V.A.04.08 (Agilent Technologies Inc., Santa Clara, CA, USA) data handling software.

### 2.3. Fecal Amino Acid Analysis

Owners were instructed to collect a voided fecal sample from their cat within 12 h of defecation and temporarily stored at 4 °C in a sealed container until sample was brought to the hospital on ice within 24 h of collection. Fecal aliquots were stored at −80 °C until analysis. After study enrollment, all fecal samples were sent to an outside laboratory (Metabolon Inc., Morrisville, NC, USA) for analysis by liquid chromatography with tandem mass spectrometry. The fecal samples were analyzed for the following 22 AAs: L-alanine, L-arginine, L-asparagine, L-aspartic acid, L-citrulline, L-glutamic acid, L-glutamine, glycine, L-histidine, hydroxy-L-proline, L-isoleucine, L-leucine, L-lysine, L-methionine, L-ornithine, L-phenylalanine, L-proline, L-serine, L-threonine, L-tryptophan, L-tyrosine, and L-valine. 

Fecal samples were lyophilized, and an aliquot was weighed. The dried fecal aliquots were then extracted with an organic solvent, and a portion of the supernatant transferred to a clean sample plate. The supernatant aliquots were spiked with stable labeled internal standards (Table S1) and subjected to protein precipitation with an organic solvent. Analyte reference materials (Sigma-Aldrich, St. Louis, MO, USA) and isotopically-labeled internal standards (Sigma-Aldrich, Cambridge Isotope Laboratories, CDN Isotopes) were obtained. After centrifugation, an aliquot of the supernatant was diluted and injected onto an Agilent 1290/AB Sciex QTrap 5500 liquid chromatography tandem mass spectrometry system equipped with a C18 reversed phase ultra-high-performance liquid chromatography column. The mass spectrometer was operated in positive mode using electrospray ionization. 

The peak areas of the individual analyte parent ions were measured against the peak areas of the parent ions of the corresponding internal standards in pseudo-MRM mode. Quantitation was performed using a weighted least squares regression analysis generated from fortified calibration standards prepared immediately prior to each run. Raw data was collected and processed using SCIEX OS-MQ software v1.7. Data reduction was performed using Microsoft Excel for Office 365 v.16. 

For the 22 analytes of the amino acid panel, a single lot of plasma was used to prepare the quality control samples. Sample analysis was carried out in a 96-well plate format containing two calibration curves and six quality control samples (per plate) to monitor assay performance. Precision was evaluated using the corresponding quality control replicates in each sample run. Intra-assay precision (%CV) was ≤20%. A single batch was prepared and processed. Concentration values were corrected based on the sample aliquot weight. Values are expressed in µg/g. The lower limit of quantification (LLOQ) for glutamine was 2.5 µg/g; for L-alanine, L-arginine, L-lysine, L-proline, L-tyrosine, and L-valine, LLOQ was 1.0 ug/g. For glycine, LLOQ was 0.75 µg/g. For L-glutamic acid, L-histidine, L-leucine, L-threonine, and L-tryptophan, LLOQ was 0.5 µg/g. For L-phenylalanine and L-serine, LLOQ was 0.4 µg/g. For L-asparagine, L-aspartic acid, L-isoleucine, and L-ornithine, LLOQ was 0.25 µg/g. For L-citrulline, LLOQ was 0.2 µg/g. For L-methionine, LLOQ was 0.125 µg/g. For hydroxy-L-proline, LLOQ was 0.05 µg/g. 

### 2.4. Statistical Analysis

For statistical comparison of results between IRIS CKD Stages, Stage 1 and 2 CKD cats and Stage 3 and 4 CKD cats were combined, given the few Stage 1 and Stage 4 CKD cats that were enrolled in the study. 

Histograms and Q-Q plots of AA measurements in healthy and CKD cats confirmed the assumption of approximate normality. Examination of boxplots showed that measurements in the CKD cats were often more variable than in the healthy cats. Thus, a Welch’s test was used to compare continuous variables between the two independent groups (i.e., healthy cats and CKD cats). A one-way Welch’s ANOVA with Holm-Sidak‘s multiple comparisons test was used to compare variables between three independent groups (i.e., healthy cats; CKD Stage 1 and 2 cats; and CKD Stage 3 and 4 cats). In addition, the Spearman correlation coefficient (rho) was computed to evaluate the correlation between serum and fecal AA concentrations and serum creatinine concentration, between serum creatinine and 3-MH concentrations, and between serum and fecal AA concentrations. The adaptive method of Benjamini, Krieger, and Yekutieli [24] was used to control for false discovery rate, with a q-value set at 5%. For statistical analysis, any samples that did not have a measurable concentration were reported as 0. When available, the absolute value was used for samples with a concentration below or above the limit of quantification. Using the reported standard deviations of plasma AA concentrations in healthy and CKD cats [9], an analysis was performed to determine the minimum difference for each AA that could be detected with a sample size in our study (16 for healthy cats; 26 CKD cats). The analysis obtained the minimum difference that could be detected for each AA with a power of 80% and alpha of 0.05. Based on the analysis, the minimum difference that could be detected is a difference of one standard deviation. Statistical analysis was performed using statistical software programs (GraphPad Prism 9.2.0; GraphPad Software, La Jolla, CA and SAS 9.4; SAS Institute, Cary, NC, USA). A corrected *p*-value < 0.05 was considered significant.

## 3. Results

### 3.1. Cats

A total of 35 cats with CKD (3 cats with IRIS Stage 1 CKD, 16 cats with IRIS Stage 2 CKD, 12 cats with IRIS Stage 3 CKD, and 4 cats with IRIS Stage 4 CKD) and 16 healthy mature adult and senior control cats were enrolled into the study. Serum AAs were measured in 26 CKD cats, and fecal AAs were measured in 31 CKD cats. Twenty-two of the 35 CKD cats had both a fecal and serum sample available for quantification of AAs. Both fecal and serum AAs were measured in all healthy cats. 

The cats with CKD (median, 14; range, 2.5–19 years) were significantly older than the healthy cats (11 (8–14 years); *p* = 0.010). The healthy cats were either a domestic short- (12/16 cats), medium- (1/16 cats), or long-haired cat (2/16 cats), with the exception of 1 cat (Himalayan). Nine were castrated males and 7 were spayed females. The cats with CKD were either a domestic short- (26/35 cats) or long-haired cat (7/35 cats), with the exception of 2 cats (1 each, Siamese and Ragdoll). Eighteen were spayed females and 17 were castrated males. 

The median BCS for healthy cats was 5.5 (range, 4–9) and that for CKD cats was 5 (range, 2–7; *p* = 0.013). The majority of CKD cats (32/35) had some degree of muscle loss based on MCS (13/32 cats had mild wasting, 15/32 cats had moderate wasting, and 4/32 cats had severe wasting). For healthy cats, 10/16 had normal muscle mass, 4/16 had mild muscle loss, and 2/16 had moderate muscle loss. 

Pertinent laboratory variables for each group (i.e., healthy cats, CKD Stages 1 and 2 cats, and CKD Stages 3 and 4 cats) are presented in Table 1. Cats with CKD had higher creatinine (*p* < 0.0001), blood urea nitrogen (*p* < 0.0001), and phosphorus (*p* = 0.042) concentrations and a lower USG (*p* < 0.0001) when compared to healthy cats. Serum albumin was not significantly different between healthy and CKD cats (*p* = 0.15). All healthy cats had a UPC less than 0.3 (range, 0.06–0.29). The majority of CKD cats (31/35; 89%) had a UPC performed, five of which showed proteinuria (range, 0.61–1.46). The four CKD cats that did not have a UPC performed were negative for protein on a urine dipstick. A serum total thyroxine concentration and absence of a thyroid slip on physical examination were used to exclude hyperthyroidism in all cats. The majority (26/35; 74%) of CKD cats received abdominal imaging (ultrasound, 25/26 CKD cats; radiographs, 1/26 CKD cats). The findings of abdominal imaging were consistent with chronic renal degenerative disease in 23/26 CKD cats but showed apparently normal renal architecture in 2/26 CKD cats, and a unilateral perinephric pseudocyst with concurrent chronic renal degenerative disease in one CKD cat. Abdominal imaging was not performed on healthy cats. 

Only 2/16 healthy cats were receiving an oral supplement (1 cat, glucosamine/chondroitin supplement and fish oil) or topical flea and tick preventative (1 cat, selamectin). Twelve of the 35 CKD cats were on one or more medications or supplements, including amlodipine (3 cats), potassium gluconate (2 cats), polyethylene glycol 3350 (3 cats), oral or injectable glucosamine chondroitin supplement (2 cats), aluminum hydroxide (2 cats), and telmisartan, fish oil, gabapentin, probiotic, transdermal mirtazapine, and topical imidacloprid and moxidectin (1 cat each). All cats were fed a commercial diet formulated to meet the Association of American Feed Control Officials Nutritional Profile for adult maintenance. For the healthy cats, 2/16 cats were fed a veterinary prescription diet formulated for weight control, 4/16 cats were fed a diet marketed for senior cats, and 10/16 cats were fed a diet marketed for adult cats. Of the 35 CKD cats, 16 were exclusively fed one or more veterinary prescription diets formulated for renal disease, 13 were exclusively fed one or more over-the-counter diets marketed for adult or senior cats, 5 cats were fed a mixture of a renal prescription diet and an over-the-counter diet, and one cat was fed a prescription diet formulated for prevention of struvite and calcium oxalate urinary stones. According to the owners, all healthy cats and 89% (31/35) CKD cats were reportedly eating 75% or more of their daily food ration.

### 3.2. Serum Amino Acids

**Figure 1:**
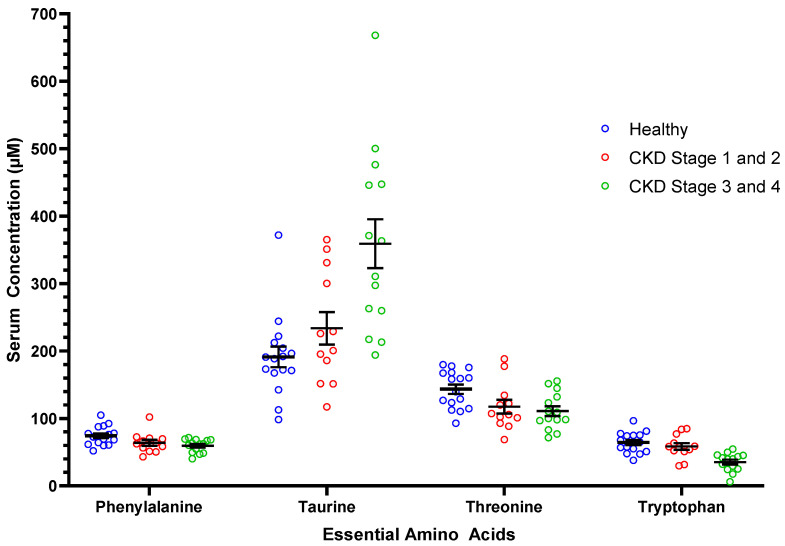
Serum concentrations of select essential amino acids in healthy mature adult and senior cats (*n* = 16), cats with CKD Stage 1 and 2 (*n* = 12), and cats with CKD Stage 3 and 4 (*n* = 14). Each dot represents a cat. The whiskers represent the mean and standard error of mean. Refer to Table 2 for statistical comparisons between groups.

Table 2 shows the AA concentrations of healthy cats, cats with CKD, and CKD cats grouped by severity of kidney disease (Stages 1 and 2; Stages 3 and 4). Compared to healthy cats, CKD cats had lower concentrations of the essential AAs phenylalanine, threonine, and tryptophan and the nonessential AAs serine and tyrosine. Cats with CKD had higher serum concentrations of the essential AA taurine and the non-essential AAs aspartic acid, β-alanine, and citrulline compared to healthy cats. Significant findings were noted for serum concentrations of six essential and six nonessential AAs when comparing between healthy cats, cats CKD Stages 1 and 2, and cats with CKD Stages 3 and 4 (Table 2). Figure 1 represents the serum concentrations of four essential amino acids (i.e., phenylalanine, taurine, threonine, tryptophan) for healthy cats, CKD Stage 1 and 2 cats, and CKD Stage 3 and 4 cats. Serum concentrations of seven essential AAs and two nonessential AAs correlated negatively with serum creatinine concentrations. Serum concentrations of four nonessential AAs correlated positively with serum creatinine concentrations (Table 3). 

### 3.3. Fecal Amino Acids

The following AAs were excluded from analysis because the fecal concentration was below the limit of quantification in >75% of the samples: arginine, asparagine, aspartic acid, glutamine, and histidine. No significant difference in fecal concentrations between CKD cats and healthy cats was found (Table S1). No fecal AA concentration significantly correlated with serum creatinine concentrations. No correlation between serum and fecal concentrations was found for the 22 amino acids that were measured in both serum and feces collected from 16 healthy cats and 22 CKD cats. 

### 3.4. Serum 3-Methylhistidine

Serum 3-MH concentrations in healthy and CKD cats are shown in Table 2. Cats with CKD had higher serum 3-MH concentrations when compared to healthy control cats (*p* = 0.005; mean difference ± standard error of the mean (SEM): 31.1 ± 8.4). Cats with CKD Stages 3 and 4 had higher serum 3-MH concentrations when compared to cats with Stage 1 and 2 CKD and healthy control cats. No difference in serum 3-MH concentrations was found between healthy cats and cats with Stage 1 and 2 CKD (*p* = 0.4). Serum 3-MH concentrations were positively correlated with serum creatinine concentrations (*p* < 0.0001; rho, 0.86; 95% confidence interval, 0.75 to 0.92). 

**Figure 2:**
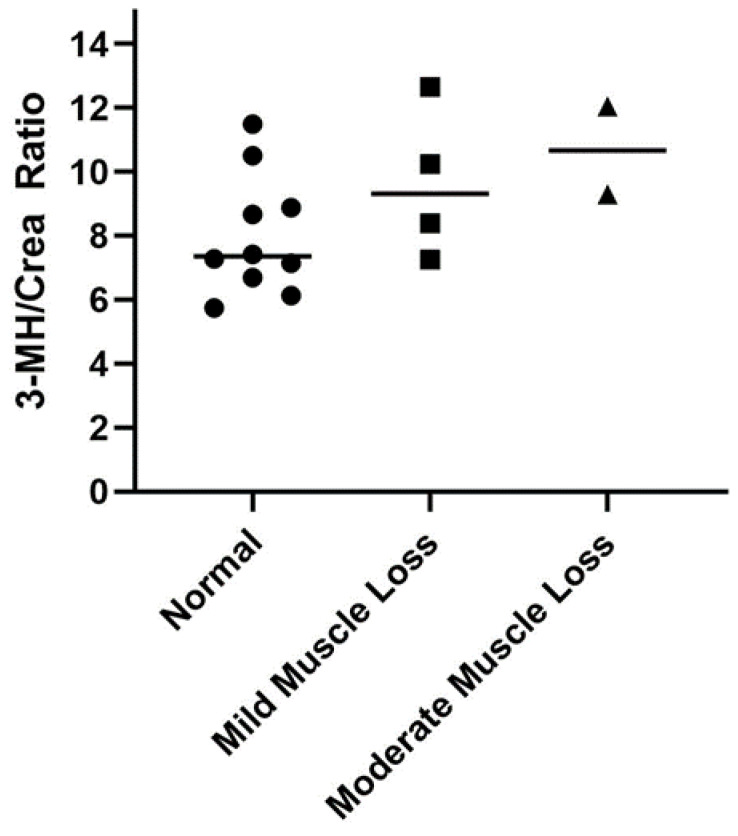
Comparison of serum 3-methylhistidine to creatinine (3-MH/Crea) ratios in healthy mature adult and senior cats (*n* = 16) separated according to muscle condition score (normal muscle mass (circle), *n* = 10; mild muscle loss (square), *n* = 4; moderate muscle loss (triangle), *n* = 2). Each point represents an individual cat, and the bar represents the mean. None of the healthy cats had severe muscle loss.

Healthy cats with mild to moderate muscle loss (*n* = 6) had higher 3-MH/Crea ratios (10.0 ± 2.1) compared to those with a normal muscle mass (*n* = 10; 8.0 ± 1.9); however, the finding was not statistically significant (*p* = 0.07; mean difference ± SEM: 2.0 ± 1.0; Figure 2).

## 4. Discussion

In this study, we measured serum and fecal AA concentrations to compare profiles between healthy mature adult and senior cats and cats with CKD. First, we found that CKD cats have a deranged serum AA profile; in particular, CKD cats had lower serum concentrations of three essential (phenylalanine, threonine, tryptophan) and two nonessential AAs (serine, tyrosine) when compared to healthy cats. Alterations in serum AA profiles occurred in cats with early-stage disease (IRIS Stage 1 and 2), and the magnitude of abnormalities was greater in cats with late-stage CKD (IRIS Stage 3 and 4) for several AAs (aspartic acid, β-alanine, citrulline, leucine, phenylalanine, serine, taurine, tryptophan, valine). Second, contrary to our hypothesis, we did not find significant differences in fecal AA concentrations between healthy cats and CKD cats. This suggests that protein malassimilation and subsequent loss of AAs in feces might not be a major contributor to the significantly deranged serum AA profile in CKD cats. Other potential causes of alterations of serum AA status in cats might include inadequate energy intake and increased protein catabolism as discussed earlier, in addition to urinary AA loss secondary to tubular dysfunction, reduced renal metabolism of select AA, and altered AA metabolism secondary to systemic inflammation [25,26]. The majority of CKD cats in this study had an adequate appetite according to the appetite scores obtained at enrollment; however, this scoring system is subjective, and calculating the daily caloric intake was not possible based on the information provided. Therefore, inadequate intake cannot be excluded as a contributing factor to the deranged AA profile in these CKD cats. Lastly, we found that 3-MH/Crea ratios did not significantly differ between healthy mature adult and senior cats with and without muscle loss. 

A previous study evaluated preprandial plasma AA concentrations in cats with CKD and showed an abnormal plasma AA profile in CKD cats compared to healthy cats [9]. Our study showed several similar differences in AA concentrations between healthy and CKD cats. Both studies showed significantly lower tryptophan and tyrosine concentrations, and higher concentrations of citrulline in CKD cats when compared to healthy cats [9]. In general, there is good reproducibility of metabolite concentrations in both plasma and serum in people; however, some AA concentrations (i.e., arginine, serine, phenylalanine, glycine) may be higher in serum [27]. Therefore, some caution should apply when directly comparing findings between our study and the previous study that measured AA concentrations in plasma.

For some AAs, serum concentrations were higher in CKD cats than in healthy cats, including the essential AA taurine and the nonessential AAs aspartic acid, citrulline, and β-alanine. Circulating concentrations of both taurine and citrulline have been found to be higher in cats and people with kidney dysfunction [9,28]. For citrulline, this finding is attributed to a reduced conversion of citrulline to arginine by the kidney [29]. Taurine is an essential amino acid in cats, unlike in people and dogs, and thus cats require dietary intake. Higher serum taurine concentrations in CKD cats might be a result of reduced renal excretion [30]. The reason for higher circulating concentrations of β-alanine and aspartic acid in CKD cats is unknown; however, this is consistent with the pattern of the circulating AA profile (i.e., high nonessential and low essential AAs) documented in people with kidney disease [28,31].

Fecal concentrations of leucine, phenylalanine, lysine, histidine, methionine, tyrosine, and tryptophan were dramatically increased in people with end-stage renal disease (ESRD) receiving hemodialysis when compared to healthy controls [15]. These undigested AAs in the colon of ESRD patients may favor proteolytic bacteria that generate the major gut-derived uremic toxins, indoxyl sulfate (IS), and *p*-cresol sulfate (pCS) [32]. People, cats, and dogs accumulate IS, while some species accumulate pCS, in the systemic circulation, and accumulation of these uremic toxins is primarily the result of reduced tubular excretion [33,34,35]. However, some theorize that protein malassimilation and increased AAs in the colon may also contribute to circulating concentrations by favoring proteolytic bacteria in the colon [6,32]. Contrary to our hypothesis, our study did not show significant differences in fecal AA concentrations between CKD and healthy cats. This puts into question the contribution of undigested AAs in the accumulation of circulating IS and pCS in CKD cats. It is possible that the lack of differences in fecal AA concentrations is due to our patient population. Most cats in our study were clinically stable, and our study cohort had too few Stage 4 CKD cats (*n* = 4) to make a meaningful comparison. It is possible that more drastic differences in the fecal AA profile might be better appreciated in cats with end-stage CKD.

We measured serum 3-MH concentrations in healthy cats and CKD cats. As expected, we found that CKD cats had higher serum 3-MH concentrations compared to healthy control cats, and that serum 3-MH concentrations positively correlated with serum creatinine concentrations. This is similar to findings of previous studies in cats, dogs, and people, which found that 3-MH concentrations in the plasma or serum of patients with CKD were higher than healthy controls [9,11,28]. This finding is attributed to reduced renal excretion of 3-MH in patients with CKD. For this reason, we did not perform the 3-MH/Crea ratio in CKD cats with muscle loss because creatinine is a confounding factor. To explore if 3-MH/Crea ratios can be used as a marker of skeletal muscle degradation in healthy mature adult and senior cats, 3-MH/Crea ratios were compared between cats with and those without muscle loss. A MCS obtained by a single veterinarian was used to assess muscle mass in this group of cats. Estimation of the MCS is easy and non-invasive for trained individuals to perform in a clinic setting and was found to have substantial repeatability and moderate reproducibility between observers [36]. Healthy cats with mild to moderate muscle loss subjectively had higher mean 3-MH/Crea ratios than those without muscle loss, but this finding was not statistically significant, which was attributed to the small sample size and potentially variable protein intakes in this study cohort [20,37]. The use of 3-MH as a biomarker of skeletal muscle degradation in cats warrants further investigation.

To combat cachexia in people with CKD, clinicians recommend increasing physical activity, treating metabolic acidosis, and eliminating correctible inflammatory factors (e.g., intestinal disease) to minimize protein catabolism. In addition, optimizing nutritional therapy is important. Similar to people, reduction in protein intake using a highly digestible protein source and maintenance of adequate caloric intake are the mainstay of treatment for cats with CKD [38,39]. A previous study in cats with IRIS Stage 1 and 2 CKD showed that enhanced intake of the essential AA threonine using a protein-restricted renal therapeutic diet (6.7 g/100 kcal) for 6 months maintained lean body mass (Hill’s Prescription Diet k/d Feline with chicken, Hill’s Pet Nutrition, Topeka, KS, USA) [40]. Amino acid profiles before and after diet transition were not evaluated in these cats. We found threonine to be low in the serum of cats with CKD compared to healthy control cats, which further supports the potential benefit of supplementation of this AA in CKD cats. In people on hemodialysis, an oral nutraceutical used to supply calories from macronutrients, including protein in the form of AAs and peptides, improved most AA concentrations in the serum [41]. Based on these studies, renal therapeutic diets fortified with essential AAs might be beneficial in CKD cats in the hope of maintaining lean body mass and potentially improving serum AA concentrations; however, the latter remains to be elucidated. 

This study has limitations. With regard to the cat populations, the control group was not age-matched to the CKD group due to the impossibility of finding healthy senior cats ˃14 years of age in the referral hospital population. As a result, the healthy control cats were significantly younger than the CKD cats, which may have affected our conclusions. In people, serum AA profiles differ between the young and elderly, and therefore, the deranged serum AA profile in CKD cats in comparison to the healthy controls may be explained in part by age [42,43]. Second, our results reflect only a single time-point and are not representative of dynamic changes that may occur in circulating or fecal AA concentrations. Third, deproteinization of serum was not performed until after freezing and just prior to analysis within 2 weeks of collection. Deproteinization prevents losing AA by co-precipitation with other serum proteins, therefore this may have impacted our results, especially for sulfur-containing AAs (methionine, taurine) [44]. However, all samples were handled similarly, so any effect should have been standardized across all samples. Lastly, all healthy cats and the majority of CKD cats (21/26) were fasted prior to blood collection. In cats, there is a mild postprandial rise in plasma AA concentrations, and the magnitude of the postprandial rise varies based on the AA concentration of the diet and feeding regimen [45,46,47]. Therefore, the fed state of these five CKD could have affected results to an unknown degree.

## 5. Conclusions

Cats with IRIS Stages 1–4 CKD have lower serum concentrations of some essential AAs when compared to healthy mature adult and senior cats, and therefore deranged serum AA profiles in CKD cats may serve as a therapeutic target of dietary management. Fecal AA concentrations were not significantly different between CKD and healthy cats, and thus fecal excretion of AAs is unlikely a major contributor to the deranged serum AA profiles documented in CKD cats. Serum 3-MH concentrations are higher in CKD cats than healthy controls, and this finding is attributed to reduced renal excretion. The 3-MH/Crea ratios were not significantly higher in healthy cats with muscle loss compared to those with normal muscle mass; however, given the small sample size further evaluation is warranted.

## Figures and Tables

**Table 1:** Cat characteristics and pertinent laboratory variables for healthy mature adult and senior cats (≥8 years), cats with IRIS CKD Stages 1 and 2, and cats with IRIS CKD Stages 3 and 4. Values provided as median and range.

Variable (Reference Interval) *	Healthy Controls (*n* = 16)	CKD Stages 1 and 2 (*n* = 19)	CKD Stages 3 and 4 (*n* = 16)
Creatinine (0.8–2.4 mg/dL)	1.4 (1.1–1.8) ^a^	2.0 (1.3–2.7) ^b^	3.7 (2.8–13.1) ^c^
BUN (18–35 mg/dL)	21 (18–29) ^a^	39 (24–49) ^b^	58 (36–117) ^c^
Phosphorus (3.0–6.0 mg/dL)	3.6 (2.9–4.6) ^a^	3.9 (2.7–5.4) ^a,b^	4.1 (3.1–7.2) ^b^
Potassium (3.7–5.4 mEq/L)	4.4 (3.6–5.2)	4.7 (3.7–5.5)	4.5 (3.5–5.1)
Albumin (3.1–4.4 g/dL)	3.8 (3.2–4.4)	3.6 (3.2–4.5)	3.6 (3.0–3.9)
USG (>1.035)	1.049 (1.038–1.056) ^a^	1.021 (1.010–1.035) ^b^	1.015 (1.006–1.023) ^c^

* For each laboratory variable, columns bearing a different superscript letter were statistically different from each other (*p* < 0.05).

**Table 2:** Serum amino acid concentrations for healthy cats, cats with CKD, and CKD cats grouped by severity of kidney failure (Stages 1 and 2; Stages 3 and 4). Effect size reported as mean difference and standard error of the mean for the comparison of serum amino acid concentrations between healthy cats and CKD cats. Amino acid concentrations provided as mean and standard deviation.

Amino Acid (µM) *	Healthy Cats (*n* = 16)	All CKD Cats (*n* = 26)	Adjusted *p*-Value (Welch *t*-Test)	Mean Difference ± SEM	CKD Stage 1 and 2 (*n* = 12)	CKD Stage 3 and 4 (*n* = 14)
Essential Amino Acids						
Arginine	115.3 ± 25.5	114.1 ± 30.0	0.63	1.2 ± 8.7	109.8 ± 25.9	117.7 ± 33.7
Histidine	107.6 ± 12.7	112.5 ± 16.5	0.35	4.9 ± 4.5	109.9 ± 15.1	114.8 ± 18.0
Isoleucine	72.5 ± 21.1	63.1 ± 24.1	0.43	9.4 ± 7.1	72.8 ± 31.1	54.7 ± 11.1
Leucine	143.1 ± 30.0 ^a^	120.4 ± 40.3	0.14	22.7 ± 10.9	135.3 ± 52.2 ^a,b^	107.5 ± 20.6 ^b^
Lysine	99.4 ± 40.7	91.2 ± 28.8	0.47	8.2 ± 11.6	100.2 ± 34.8	83.5 ± 20.8
Methionine	46.2 ± 15.6	37.9 ± 20.7	0.33	8.3 ± 5.6	39.4 ± 24.1	36.7 ± 18.1
Phenylalanine	74.3 ± 13.9 ^a^	61.6 ± 12.4	0.03	12.7 ± 4.3	64.0 ± 15.0 ^a,b^	59.6 ± 9.8 ^b^
Taurine	191.3 ± 61.1 ^a^	301.2 ± 129.2	0.01	109.9 ± 29.6	233.7 ± 83.6 ^a^	359.1 ± 135.5 ^b^
Threonine	143.3 ± 28.0 ^a^	113.9 ± 30.5	0.03	29.6 ± 9.2	117.4 ± 35.0 ^b^	110.8 ± 27.1 ^b^
Tryptophan	64.4 ± 15.2 ^a^	46.1 ± 19.2	0.005	18.4 ± 5.4	58.5 ± 17.5 ^a^	35.4 ± 13.5 ^b^
Valine	179.5 ± 41.2 ^a^	151.9 ± 52.3	0.19	27.7 ± 14.5	172.5 ± 66.6 ^a,b^	134.2 ± 27.8 ^b^
Nonessential Amino Acids						
Alanine	580.2 ± 116.2	545.8 ± 108.2	0.38	34.3 ± 36.0	531.6 ± 110.9	558.0 ± 108.4
Asparagine	100.7 ± 25.1	85.0 ± 23.4	0.19	15.7 ± 7.8	85.4 ± 25.0	84.8 ± 22.9
Aspartic acid	18.7 ± 5.5 ^a^	23.4 ± 7.6	0.005	4.7 ± 2.0	18.3 ± 4.9 ^a^	27.7 ± 6.8 ^b^
β-alanine	3.2 ± 3.5 ^a^	6.6 ± 3.8	0.005	3.4 +/1.2	4.1 ± 2.4 ^a^	8.8 ± 3.5 ^b^
Citrulline	12.2 ± 4.0 ^a^	17.9 ± 6.2	0.01	5.7 ± 1.6	15.0 ± 3.9 ^a^	20.4 ± 6.8 ^b^
Glutamic acid	38.0 ± 17.8	34.1 ± 12.1	0.30	3.9 ± 5.0	37.2 ± 14.0	31.5 ± 9.9
Glutamine	678.8 ± 98.0	701.7 ± 176.4	0.34	22.9 ± 42.4	661.9 ± 159.4	735.9 ± 188.8
Glycine	308.6 ± 61.6	308.2 ± 82.5	0.89	0.4 ± 22.3	300.5 ± 71.1	314.8 ± 93.3
Hydroxyproline	28.8 ± 15.0	34.1 ± 18.6	0.52	5.0 ± 5.1	29.9 ± 15.7	37.1 ± 20.6
Ornithine	14.6 ± 4.9	13.0 ± 4.3	0.57	1.5 ± 1.5	12.8 ± 4.8	13.2 ± 4.0
Proline	194.9 ± 70.8	145.6 ± 41.2	0.10	49.3 ± 19.5	143.6 ± 50.6	147.3 ± 33.0
Serine	146.4 ± 35.5 ^a^	116.6 ± 45.4	0.03	29.8 ± 12.6	132.6 ± 48.2 ^a,b^	103.0 ± 39.5 ^b^
Tyrosine	55.1 ± 13.4 ^a^	43.5 ± 9.0	0.01	11.6 ± 3.8	41.5 ± 10.0 ^b^	45.3 ± 8.0 ^b^
3-Methylhistidine	11.6 ± 2.8 ^a^	42.7 ± 42.5	0.005	31.1 ± 8.4	27.3 ± 13.5 ^a^	55.8 ± 53.9 ^b^

* For the statistical comparison between healthy cats, CKD Stages 1 and 2 cats, and CKD Stages 3 and 4 cats; columns bearing a different superscript letter were significantly different from one another (*p* < 0.05).

**Table 3:** Spearman rank correlation coefficients and *p*-value for significant correlations found between serum concentrations of essential and nonessential amino acids and serum creatinine concentrations.

Serum Amino Acid	Spearman’s Rank Correlation Coefficient (95% Confidence Interval (Upper Limit, Lower Limit)	Adjusted *p*-Value
Essential Amino Acids		
Isoleucine	−0.36 (−0.60, −0.05)	0.02
Leucine	−0.45 (−0.67, −0.16)	0.005
Methionine	−0.39 (−0.63, −0.10)	0.01
Phenylalanine	−0.44 (−0.66, −0.15)	0.005
Threonine	−0.39 (−0.63, −0.10)	0.01
Tryptophan	−0.58 (−0.76, −0.33)	<0.001
Valine	−0.45 (−0.67, −0.16)	0.005
Nonessential Amino Acids		
Aspartic acid	0.68 (0.47, 0.82)	<0.001
β-alanine	0.58 (0.32, 0.75)	<0.001
Citrulline	0.49 (0.20, 0.69)	0.002
Serine	−0.43 (−0.66, −0.14)	0.005
Taurine	0.67 (0.46, 0.81)	<0.001
Tyrosine	−0.33 (−0.58, −0.02)	0.03

## Data Availability

The data presented in this study are available on request from the corresponding author.

## Supplementary Materials

The following supporting information can be downloaded at: https://www.mdpi.com/article/10.3390/vetsci9020084/s1, Table S1: Fecal amino acid concentrations for healthy cats and cats with chronic kidney disease (CKD).
