# Supplementary material for: Serum and Fecal Amino Acid Profiles in Cats with Chronic Kidney Disease
Source: Vet Sci. 2022 Feb 17;9(2):84. doi: 10.3390/vetsci9020084 (PMC8878831; doi:10.3390/vetsci9020084)
Supplement: Supplementary file 1 [file vetsci-09-00084-s001.zip › vetsci-1587056-supplementary.pdf]

veterinary sciences

MDPI

Supplementary Materials

Serum and Fecal Amino Acid Profiles in Cats with Chronic Kidney Disease

Stacie C. Summers, Jessica Quimby, Amanda Blake, Deborah Keys, Joerg M. Steiner and Jan Suchodolski

Table S1. Fecal amino acid concentrations for healthy cats and cats with chronic kidney disease (CKD). Effect size reported as mean difference and standard error of the mean (SEM).

Amino Acid (µg/g)	Healthy cats (n=16)	CKD Cats (n=31)	Unadjusted P-value (Welch t-test)	Mean Difference +/- SEM
Essential Amino Acids				
Isoleucine	24.0 +/- 17.0	25.8 +/- 16.4	0.74	1.7 +/- 5.2
Leucine	38.9 +/- 25.0	37.7 +/- 21.9	0.87	1.2 +/- 7.4
Lysine	5.9 +/- 6.9	8.5 +/- 8.0	0.27	2.5 +/- 2.2
Methionine	8.3 +/- 5.7	9.9 +/- 5.8	0.37	1.6 +/- 1.8
Phenylalanine	18.3 +/- 11.8	17.8 +/- 10.9	0.90	0.4 +/- 3.5
Threonine	4.2 +/- 3.1	5.7 +/- 4.2	0.20	1.4 +/- 1.1
Tryptophan	5.5 +/- 4.4	6.3 +/- 4.3	0.57	0.8 +/- 1.3
Valine	25.7 +/- 17.8	28.1 +/- 18.2	0.66	2.5 +/- 5.5
Nonessential Amino Acids				
Alanine	23.6 +/- 16.7	27.6 +/- 18.8	0.46	4.0 +/- 5.4
Aspartic acid	0.28 +/- 0.6	0.7 +/- 0.7	0.12	0.3 +/- 0.2
Citrulline	5.8 +/- 4.6	7.9 +/- 6.2	0.20	2.1 +/- 1.6
Glutamic acid	2.5 +/- 2.4	4.1 +/- 4.3	0.10	1.6 +/- 1.0
Glycine	1.3 +/- 1.2	2.5 +/- 2.7	0.03	1.2 +/- 0.6
Hydroxyproline	0.70 +/- 0.94	0.52 +/- 0.72	0.51	0.18 +/- 0.27
Ornithine	0.35 +/- 0.46	0.54 +/- 0.55	0.23	0.19 +/- 0.15
Proline	6.9 +/- 4.5	11.6 +/- 9.8	0.03	4.7 +/- 2.1
Serine	1.3 +/- 1.3	1.6 +/- 1.6	0.44	0.33 +/- 0.42
Tyrosine	10.6 +/- 6.6	14.7 +/- 9.9	0.10	4.1 +/- 2.4
